# Anal carcinoma in inflammatory bowel disease

**DOI:** 10.1054/bjoc.2000.1153

**Published:** 2000-06-02

**Authors:** M Frisch, C Johansen

**Affiliations:** Department of Epidemiology Research, Danish Epidemiology Science Centre, Statens Serum Institut, 5 Artillerivej, Copenhagen, DK-2300, Denmark; Danish Cancer Society, Institute of Cancer Epidemiology, 49 Strandboulevarden, Copenhagen, DK-2100, Denmark

**Keywords:** anus neoplasms, ulcerative colitis, Crohn's disease, inflammatory bowel disease, cohort studies

## Abstract

We followed 9602 patients with Crohn's disease or ulcerative colitis for anal squamous cell carcinoma for up to 18 years. No significant increase was observed: two cases occurred *vs* 1.3 expected during 99 229 person-years of observation, (standardized incidence ratio = 1.6; 95 confidence interval: 0.2–5.7). Anal squamous cell carcinoma is rare even in inflammatory bowel disease. © 2000 Cancer Research Campaign


*British Journal of Cancer* (2000) **83**(1), 89–90
© 2000 Cancer Research Campaign
doi: 10.1054/ bjoc.2000.1153, available online at http://www.idealibrary.com on IDEAL®

Short communication

### Anal carcinoma in inflammatory bowel disease

**M Frisch[1] and C Johansen[2]**

[1] Department of Epidemiology Research, Danish Epidemiology Science Centre, Statens Serum Institut, 5 Artillerivej, DK-2300 Copenhagen, Denmark; [2]Danish Cancer Society, Institute of Cancer Epidemiology, 49 Strandboulevarden, DK-2100 Copenhagen, Denmark.

**Summary** We followed 9602 patients with Crohn's disease or ulcerative colitis for anal squamous cell carcinoma for up to 18 years. No significant increase was observed: two cases occurred *vs* 1.3 expected during 99 229 person-years of observation, (standardized incidence ratio = 1.6; 95 confidence interval: 0.2–5.7). Anal squamous cell carcinoma is rare even in inflammatory bowel disease. © 2000 Cancer Research Campaign

**Keywords:** anus neoplasms; ulcerative colitis; Crohn's disease; inflammatory bowel disease; cohort studies

For decades anal inflammation, a common problem in patients with inflammatory bowel disease (IBD), has been suspected to predispose for anal squamous cell carcinoma (SCC) (Brofeldt, 1927). We previously examined the risk of anal SCC among patients with haemorrhoids, fissures, fistulae, or abscesses and failed to support a causal relationship up to 13 years after those benign anal lesions (Frisch et al, 1994). Also, no convincing association was seen between IBD and the risk of anal SCC in the only case-control study addressing the question (Frisch et al, 1998). Using Danish health registers, we generate a population-based estimate of the incidence of anal SCC in patients with IBD and compare this estimate with the incidence of anal SCC in the general population.

### MATERIAL AND METHODS

We identified 9875 Danish patients with valid personal identifiers who were hospitalized during the period 1977–1989 with a diagnosis of either Crohn's disease (ICD8: 563.01) or ulcerative colitis (ICD8: 563.19). Forty-one patients with polyposis coli (ICD8: 211.36) and 232 patients who died during the first hospitalization for IBD were excluded, leaving 9602 IBD patients for follow-up. We assessed the risk of anal SCC among patients with unambiguous diagnoses of either Crohn's disease (1067 men, 1656 women) or ulcerative colitis (2988 men, 3346 women). To evaluate the overall risk of anal SCC among IBD patients, we combined the two cohorts and included an additional 505 patients registered with both Crohn's disease and ulcerative colitis, and 40 patients with both Crohn's disease and proctitis (ICD8: 569.04).

Person-years were counted from the date of first hospital discharge after the cohort defining IBD until anal SCC, death, or January 1, 1995, whichever came first. Expected incidence rates of anal SCC were calculated as weighed population incidence rates of anal SCC (Frisch et al, 1993), with weights reflecting the gender, age, and calendar period composition of the IBD cohorts. Observed incidence rates were calculated directly as the observed number of anal SCCs divided by the person-years. Ratios of observed to expected anal SCC incidence per 100 000 person-years (with 95% confidence intervals (CIs)) estimated relative risk (RR).

*Received 4 January 2000*
*Revised 7 February 2000*
*Accepted 7 February 2000*

*Correspondence to:* M Frisch

### RESULTS

2723 Crohn's disease patients were followed 27 647 person-years for anal SCC; mean follow-up = 10.2 (range 0–18) years. Mean age at first hospitalization was 39.9 years. Fifteen years after her first registered hospitalization for Crohn's disease and 41 years after a cervical cancer, one woman developed anal SCC at age 80 years (observed incidence = 3.62 per 100 000, expected incidence = 1.21 per 100 000, RR = 3.0; 95% CI: 0.0–16.7).

6334 ulcerative colitis patients were followed 65 281 person-years; mean follow-up = 10.3 (range 0–18) years. Mean age at first hospitalization was 43.1 years. One woman developed anal SCC at age 75 years, 16 years after her first registered hospitalization for ulcerative colitis (observed incidence = 1.53 per 100 000, expected incidence = 1.34 per 100 000, RR = 1.1; 95% CI: 0.0–6.4).

The combined cohort of 9602 IBD patients was followed for 99 229 person-years. Only the two patients mentioned above developed anal SCC (RR = 1.6; 95% CI: 0.2–5.7).

### DISCUSSION

The two cases of anal SCC occurred 15 and 16 years after the onset of IBD symptoms. If inflammatory bowel diseases play a causal role in a few cases of anal SCC, this may be restricted to patients with long-standing Crohn's disease or ulcerative colitis. Although we followed a large cohort of patients for up to 18 years, few patients had follow-up periods longer than 15 years. Consequently, our data do not exclude a long-term effect (Connell et al, 1994).

In the general Danish population, anal SCCs occur at an incidence of around 0.5–1.0 per 100 000 person-years (Frisch et al, 1993). Our observation of two anal SCCs during approximately

99 000 person-years suggests a slightly increased risk of anal SCC in IBD patients with disease of long duration. However, with an incidence of approximately one case of anal SCC per 5000 inflammatory bowel disease patients followed for 10 years, anal SCC is a rare and not significantly increased malignancy even in this group.

### ACKNOWLEDGEMENT

Thanks to programmer Anne-Marie Egelund Larsen for computer assistance.

### REFERENCES

Brofeldt SA (1927) Zur Pathogenese des Plattenepithelkrebses der Pars analis recti. *Acta Soc Med Fenn Duodecim* **8**: 3–15

Connell WR, Sheffield JP, Kamm MA, Ritchie JK, Hawley PR and Lennard-Jones JE (1994) Lower gastrointestinal malignancy in Crohn's disease. *Gut* **35**: 347–352

Frisch M, Melbye M and Møller H (1993) Trends in incidence of anal cancer in Denmark. *BMJ* **306**: 419–422

Frisch M, Olsen JH, Bautz A and Melbye M (1994) Benign anal lesions and the risk of anal cancer. *N Engl J Med* **331**: 300–302

Frisch M, Glimelius B, van den Brule AJ, Wohlfahrt J, Meijer CJ, Walboomers JM, Adami HO and Melbye M (1998) Benign anal lesions, inflammatory bowel disease and risk for high-risk human papillomavirus-positive and -negative anal carcinoma. *Br J Cancer* **78**: 1534–1538

*© 2000 Cancer Research Campaign*

